# RETRACTION: Comprehensive Analysis of Risk Factors and Pathogenetic Characteristics Associated With Surgical Site Infections Following Craniotomy Procedures

**DOI:** 10.1111/iwj.70572

**Published:** 2025-04-18

## Abstract

**RETRACTION:** GuZ. , TuC. , SongD. , YangZ. , and XiaJ. , “Comprehensive Analysis of Risk Factors and Pathogenetic Characteristics Associated With Surgical Site Infections Following Craniotomy Procedures,” International Wound Journal 21, no. 4 (2024): e14550, 10.1111/iwj.14550.38069518 PMC10961042

The above article, published online on 08 December 2023, in Wiley Online Library (http://onlinelibrary.wiley.com/), has been retracted by agreement between the journal Editor in Chief, Professor Keith Harding; and John Wiley & Sons Ltd. Following an investigation by the publisher, all parties have concluded that this article was accepted solely on the basis of a compromised peer review process. The editors have therefore decided to retract the article. The authors did not respond to our notice regarding the retraction.

**RETRACTION:** Z. Gu , C. Tu , D. Song , Z. Yang , and J. Xia , “Comprehensive Analysis of Risk Factors and Pathogenetic Characteristics Associated With Surgical Site Infections Following Craniotomy Procedures,” International Wound Journal 21, no. 4 (2024): e14550, 10.1111/iwj.14550.38069518 PMC10961042

The above article, published online on 08 December 2023, in Wiley Online Library (http://onlinelibrary.wiley.com/), has been retracted by agreement between the journal Editor in Chief, Professor Keith Harding; and John Wiley & Sons Ltd. Following an investigation by the publisher, all parties have concluded that this article was accepted solely on the basis of a compromised peer review process. The editors have therefore decided to retract the article. The authors did not respond to our notice regarding the retraction.

